# Supplementary material for: Adaptation of the PERCEPT myeloma prehabilitation trial to virtual delivery: changes in response to the COVID-19 pandemic
Source: BMJ Open. 2022 Apr 8;12(4):e059516. doi: 10.1136/bmjopen-2021-059516 (PMC8995568; doi:10.1136/bmjopen-2021-059516)
Supplement: Author's manuscript [file bmjopen-2021-059516.draft_revisions.pdf]

BMJ Open

BMJ Open is committed to open peer review. As part of this commitment we make the peer review history of every article we publish publicly available.

When an article is published we post the peer reviewers' comments and the authors' responses online. We also post the versions of the paper that were used during peer review. These are the versions that the peer review comments apply to.

The versions of the paper that follow are the versions that were submitted during the peer review process. They are not the versions of record or the final published versions. They should not be cited or distributed as the published version of this manuscript.

BMJ Open is an open access journal and the full, final, typeset and author-corrected version of record of the manuscript is available on our site with no access controls, subscription charges or pay-per-view fees (http://bmjopen.bmj.com).

If you have any questions on BMJ Open's open peer review process please email info.bmjopen@bmj.com

BMJ Open

Adaptation of the PERCEPT myeloma prehabilitation trial to virtual delivery: changes in response to the COVID-19 pandemic

Journal:	*BMJ Open*
Manuscript ID	bmjopen-2021-059516.R1
Article Type:	Communication
Date Submitted by the Author:	01-Dec-2021
Complete List of Authors:	McCourt, Orla; University College London Hospitals NHS Foundation Trust, ; University College London, Cancer Institute<br>Fisher, Abigail; University College London, Department of Epidemiology and Public Health<br>Ramdharry, Gita; National Hosital for Neurology and Neurosurger, Queen Square Centre for Neuromuscular Diseases; UCL, Deprtment of Neuromuscular Diseases<br>Land, Joanne; University College London, Behavoural Science and Health<br>Roberts, Anna; University College London, Behavoural Science and Health<br>Rabin, Neil; University College London Hospitals NHS Foundation Trust, Department of Haematology<br>Rowe, Katie; Univesrsity College London<br>Yong, Kwee; Univesrsity College London, Research Department of Haematology, Cancer Institute
**Primary Subject Heading**:	Haematology (incl blood transfusion)
Secondary Subject Heading:	Rehabilitation medicine
Keywords:	Myeloma < HAEMATOLOGY, Bone marrow transplantation < HAEMATOLOGY, COVID-19, Rehabilitation medicine < INTERNAL MEDICINE

SCHOLARONE™
Manuscripts

I, the Submitting Author has the right to grant and does grant on behalf of all authors of the Work (as defined in the below author licence), an exclusive licence and/or a non-exclusive licence for contributions from authors who are: i) UK Crown employees; ii) where BMJ has agreed a CC-BY licence shall apply, and/or iii) in accordance with the terms applicable for US Federal Government officers or employees acting as part of their official duties; on a worldwide, perpetual, irrevocable, royalty-free basis to BMJ Publishing Group Ltd ("BMJ") its licensees and where the relevant Journal is co-owned by BMJ to the co-owners of the Journal, to publish the Work in BMJ Open and any other BMJ products and to exploit all rights, as set out in our licence.

The Submitting Author accepts and understands that any supply made under these terms is made by BMJ to the Submitting Author unless you are acting as an employee on behalf of your employer or a postgraduate student of an affiliated institution which is paying any applicable article publishing charge ("APC") for Open Access articles. Where the Submitting Author wishes to make the Work available on an Open Access basis (and intends to pay the relevant APC), the terms of reuse of such Open Access shall be governed by a Creative Commons licence – details of these licences and which Creative Commons licence will apply to this Work are set out in our licence referred to above.

Other than as permitted in any relevant BMJ Author's Self Archiving Policies, I confirm this Work has not been accepted for publication elsewhere, is not being considered for publication elsewhere and does not duplicate material already published. I confirm all authors consent to publication of this Work and authorise the granting of this licence.

For peer review only

Note from the Editors: Instructions for reviewers of communication articles

The communication article is a new article type considered by BMJ Open for submissions that do not meet BMJ Open's criteria for a research article but are of interest and relevance to the journal's wide, international readership. The content can be any area of medical research that directly addresses patient outcomes or the practice and delivery of healthcare. Examples include: explanation and elaboration of research reporting guidelines; initiatives aiming to improve data sharing practices; brief reports on the development and evaluation of complex interventions, and; initiatives to reduce research waste and identify priority areas for medical research.

When reviewing this manuscript please consider the following points:

- Are the issues raised by the article important to BMJ Open's broad and international readership that includes patients, researchers, policy makers, health professionals, and doctors of all disciplines?

- Is the article interesting and offering novel insights that have not been sufficiently considered in the existing published literature?

- Is the article well written and is the content clearly presented? Does it have a clear message?

- Will the article help medical researchers, patients or related groups of readers to make better decisions?

- Does the article demonstrate one or more of the following values: transparency, openness, collaboration, innovation, reproducibility, patient/ public involvement, improving peer review and journal best practice, and reducing research waste?

Some additional things to keep in mind when reviewing communication articles:

- Unfortunately, we are unable to customize the reviewer report form for communication articles. As such, most of the items on the form should be scored as Not Applicable (N/A).
- BMJ Open will occasionally consider commentaries, opinion pieces and debates submitted under this new article type.
- Communication articles can include methods, results and discussion sections, if applicable, but these sections are not compulsory.

For peer review only

Adaptation of the PERCEPT myeloma prehabilitation trial to virtual delivery: changes in response to the COVID-19 pandemic

Corresponding Author:

Orla McCourt

Department of Haematology, UCL Cancer Institute, University College London, UK & University College London Hospitals NHS Foundation Trust, London, UK

Postal Address: Therapy Office, Lower Ground Floor (T-1), University College Hospital, 235 Euston Road, London NW1 2BU, UK

Email: o.mccourt@nhs.net

Telephone: 020 7679 1736

Authors:

Orla McCourt – Therapies & Rehabilitation, University College London Hospitals NHS Foundation Trust, London, UK; Research Department of Haematology, Cancer Institute, University College London, London, UK

Abigail Fisher - Research Department of Behavioural Science and Health, University College London, London, UK

Gita Ramdharry - Queen Square MRC Centre for Neuromuscular Diseases, Institute of Neurology, University College London, London, UK

Anna Roberts - Research Department of Behavioural Science and Health, University College London, London, UK

Joanne Land - Research Department of Haematology, Cancer Institute, University College London, London, UK

Neil Rabin – Department of Haematology, University College London Hospitals NHS Foundation Trust, London, UK

Katie Rowe - Research Department of Haematology, Cancer Institute, University College London, London, UK

Kwee Yong - Research Department of Haematology, Cancer Institute, University College London, London, UK

Word Count: 1810

For peer review only

ABSTRACT

Introduction and objective Research activity was impacted by the novel coronavirus disease (COVID-19) pandemic, the PERCEPT myeloma trial was no exception. This pilot randomised trial delivered a face-to-face exercise intervention prior to and during autologous stem cell transplantation (ASCT) in myeloma patients, as a consequence of COVID-19 it required significant adaptions to continue. This brief communication describes how the previously published study protocol was adapted for virtual delivery. In addition, we highlight the challenge of continuing the study which was embedded within a clinical pathway also impacted by the pandemic.

Summary The original trial protocol was amended and continued to recruit and deliver an exercise prehabilitation intervention virtually. Continued delivery of the intervention was deemed important to participants already enrolled within the trial and the adapted virtual version of the trial was acceptable to the research ethics committee as well as participants. Development of effective, remotely delivered rehabilitation and physical activity programmes are likely to benefit people living with myeloma. The COVID-19 pandemic provided an opportunity to explore the feasibility of a programme for ASCT recipients, however continued changes to the clinical pathway upon which the study was embedded posed the greatest challenge.

STRENGTHS AND LIMITATIONS OF THIS STUDY

- Challenges arising from the emergence of the COVID-19 pandemic offered the opportunity to extend this feasibility study to explore virtual delivery of the exercise prehabilitation intervention as well as study recruitment and assessments.
- Virtual delivery of the study was acceptable to research ethics committee and participants.
- Continued alterations to the clinical pathway upon which the study was designed posed the greatest threat to the study’s primary aim.
- Development and feasibility testing of virtually delivered rehabilitation and physical activity interventions are important for people with haematological cancers, for whom periods of immunosuppression and recovery from treatment will continue to be challenging beyond the COVID-19 pandemic.

For peer review only

INTRODUCTION

The threat of the novel coronavirus disease (COVID-19) and declaration of a pandemic greatly impacted research activity around the world with many studies and trials paused and recruitment ceased unless COVID-19 related or providing potential life preserving interventions[1]. Pandemic responses led to changes in how clinical services were delivered across health systems, with many usual face to face outpatient activities changing quickly to be remotely delivered via telephone or video.

Reports have detailed how clinical services and clinical trials adapted in response to the need to lower the threat posed by COVID-19, initially through reduced attendance at healthcare institutions and protecting the most clinically vulnerable by promoting minimal activity outside of the home, practising social distancing and discouraging use of public transport. In the United Kingdom, people considered to be clinically extremely vulnerable, such as those with haematological cancers, were advised to shield to protect themselves from exposure to infection. As a result, clinical pathways and research protocols required significant adaptations to continue[2-4]. We report the challenges of continuing and adapting a prehabilitation and rehabilitation exercise intervention trial in response to the COVID-19 outbreak and subsequent changes to the clinical pathway into which the trial was integrated. This communication seeks to document changes to our previously published protocol, as well as facilitate study design in the immediate post pandemic era where future proofing interventional trials to adapt to future pandemic or health emergencies may need to be considered.

BACKGROUND TO TRIAL

The PERCEPT myeloma prehabilitation study was a pilot, single-centre, randomised controlled trial (RCT) that aimed to investigate the feasibility of a physiotherapist-led exercise intervention, designed to be embedded as an integral part of an established clinical pathway for patients with multiple myeloma undergoing autologous stem cell transplantation (ASCT). Recruitment to the trial commenced at University College Hospital London (UCLH) in June 2019. The original trial was registered (ISRCTN15875290) and the protocol is described in detail elsewhere[5]. In summary the trial assessments, which included a range of objective measures of functional capacity as well as patient reported outcome measures (PROMs), were initially designed to be conducted face-to-face at established touchpoints within the clinical pathway that usually coincided with patient attendance at transplant clinic appointments. The intervention involved an exercise intervention with incorporated behaviour change techniques, delivered in part through weekly face-to-face, group-based exercise sessions at a UCLH gym supervised by a physiotherapist and through unsupervised home exercise sessions with telephone support in the post-ASCT rehabilitation phase.

For peer review only - http://bmjopen.bmj.com/site/about/guidelines.xhtml

TRIAL RECRUITMENT PRIOR TO THE PANDEMIC

Trial recruitment was progressing as planned and between June 2019 and end of February 2020 90 potential participants had been identified and screened for eligibility. Of those, n=36 (40%) consented to take part and were randomised, n=8 (9%) were ineligible and n=46 (51%) declined. The main reasons for declining to take part were travel or distance to take part in the intervention, poor mobility/inability to use public transport and already having too many hospital appointments. Many of those who declined had a favourable opinion of the trial and would have taken part if it was closer to their home. It had been anticipated that target recruitment (n=60-75) would be reached by December 2020.

Face to face recruitment was paused in March 2020, due to the COVID-19 pandemic, when all but essential NHS research activity was instructed to cease and research physiotherapists were redeployed to clinical services. In addition, the myeloma ASCT service was also paused due to resource limitations, lack of critical care provision and increased risk of COVID-19 infection in transplant recipients.

RESUMING RECRUITMENT AND ADAPTATION TO VIRTUAL INTERVENTION DELIVERY

Between March and June 2020, all participants already enrolled within the trial expressed their wish to continue in the trial whilst awaiting their delayed ASCT. All participants were placed on holding chemotherapy by their clinical teams. A minor ethics amendment was granted to allow participants to remain in the trial longer than in the original protocol, to continue delivery of the exercise intervention via telephone support and to collect follow up assessment data via postal delivery of trial questionnaires.

Following resumption of the myeloma ASCT clinical service in June 2020, elements of the clinical pathway on which the trial procedures were designed had changed significantly in response to the ongoing threat of the pandemic. Namely, patients were no longer attending UCLH clinics in person in the lead up to their ASCT and were instead receiving telephone contact for clinic appointments. It was therefore necessary to adapt the trial to accommodate these changes to the clinical pathway.

Patient and public involvement

Participants who had remained in the study during the first national lock down were consulted on how best to proceed with the trial. These participants were asked to consider their acceptability of receiving study assessments/questionnaires via post, and importantly, how they would feel about only having access to the physiotherapist and exercise intervention via video platform. Overwhelmingly, they supported adapting the trial to virtual delivery and reported that having regular contact with the

For peer review only

study physiotherapist was supportive, especially as they were no longer attending their clinical services in person. Some reported that the exercise intervention was something positive to focus on while feeling apprehensive about having their ASCT delayed and remaining limited in their social activities due to shielding. These participants were consulted on the proposed substantial amendment and gave their approval to the changes described in this manuscript.

Ethical approval

Following review of emerging literature for adapting pulmonary rehabilitation services to virtual delivery[6-8] and liaising with other cancer rehabilitation services delivering exercise virtually, the trial design was substantially amended. A substantial amendment was approved by the ethics committee (London - Camden & Kings Cross reference 19/LO/0204) to continue the trial with virtual enrolment and consent process, baseline assessment and delivery of a group-based intervention via the online platform Zoom, described below.

Virtual recruitment, consenting and baseline assessments

- Identifying potential participants from a list of patients awaiting consideration for ASCT, rather than from weekly clinic lists of expected clinic attendance.
- An additional inclusion criterion was added to require patients to have access to and the ability to use internet-based video conferencing platform, or a family member to facilitate access.
- Trial consent forms, self-complete questionnaire measures and pre-programmed ActivPAL accelerometer were posted to participant with a Zoom video call appointment arranged for approximately 3 days later. Completion of consent forms, baseline questionnaires and fitting of accelerometer discussed and conducted virtually over Zoom appointment.
- Addition of the Activities-Specific Balance Confidence Scale[9] to assess risk of falling, used to screen those randomised to the intervention for any necessary actions required to reduce risk of exercising at home as no longer objectively assessed by the physiotherapist face to face.
- Objective measure of functional capacity changed to a remotely delivered one minute sit to stand test, which has been demonstrated to induce comparable cardiovascular stress and lower limb muscle fatigue as the six minute walk test[10].

Virtual delivery of the partly supervised exercise intervention

- The intervention delivery was changed to be completely online – with an initial one to one session with each new participant to introduce the programme and tailor to their individual abilities, including any symptoms of bone disease or balance deficits. Each participant was

For peer review only

then asked to attend a weekly virtual group-based exercise class via Zoom until they were admitted for their ASCT.

RECRUITMENT TO VIRTUAL TRIAL TO DATE

Recruitment restarted after the first wave of the pandemic in August 2020. Up to October 2020 a further 33 potential participants were identified and screened for eligibility. Of those, 14 (43%) agreed and consented to take part, 6 (18%) were ineligible and 13 (39%) declined. This indicates that uptake to the virtual part of the trial was similar to the original face to face design. Despite fewer people declining the virtual trial, more people were ineligible. The main reason for increased proportion of ineligibility was related to some potential participants already having a date to be admitted for ASCT within the succeeding 2-4 weeks or not having access to the internet.

With the resurgence of COVID-19 in the Autumn of 2020 and as clinical services for ASCT were disrupted again, it was decided to cease recruitment to the trial. The predominant challenge to the adapted trial was variable intervals between timepoints, which differed in length from those detailed in the original study schedule. Further, a small number of participants were withdrawn from the trial as their planned ASCT was deferred or delayed due to clinical risk of proceeding with the ongoing threat of the COVID-19 virus and prioritisation of ASCT admissions based on clinical need in this context. The main aim of the study was to investigate the feasibility of delivering an intervention embedded within an existing clinical pathway. As the pathway upon which the trial was designed was significantly affected by the Covid-19 pandemic, which would likely continue to affect the pathway for some time, it was agreed by the study team that little more would be gained by recruiting further. The trial continued in follow up until all those recruited either reached the final timepoint following ASCT or were withdrawn. The challenges to timely recruitment and delivery of the trial timepoints as based on the clinical ASCT pathway have implications on study design and will be addressed with the full reporting of the trial which is expected in early 2021.

CONCLUSION

The emergence of the COVID-19 pandemic required significant adaptation for the continuation of this pilot RCT. Our sample population being clinically extremely vulnerable were advised to shield, as well as the requirement to reduce all but clinically necessary face to face contact altered how potential ASCT recipients accessed the clinical setting and consequently the planned trial delivery. We have briefly described how the trial protocol was amended and continued to recruit to and deliver a prehabilitation intervention virtually. Adaptation to virtual delivery was deemed important to participants already enrolled within the trial and appears to have been acceptable for the adapted virtual version of the trial while this was possible. As ASCT recipients with myeloma are generally older,

For peer review only

with high incidence of disease-related bone disease, deconditioned by induction chemotherapy with possible impairments to mobility and function, adapting the trial to explore feasibility of a virtually delivered physiotherapy intervention was an important opportunity arising from the threat posed by the pandemic. Considering the possible increased risk from not assessing participants face to face, required implementation of a remotely delivered balance assessment tool, an alternate objective measure of functional capacity suitable for remote assessment, and on-line adaptation of intervention delivery to include a virtual group-based exercise class were all important steps. Development of effective, remotely delivered rehabilitation and physical activity programmes are likely to be increasingly valuable to people living with myeloma for whom shielding during clinically vulnerable periods of treatment and recovery, and travel to their specialist treatment centres will be continuing challenges in the post pandemic era.

FUNDING STATEMENT

This communication relates to independent research supported by the National Institute for Health Research (HEE/ NIHR ICA Programme Clinical Doctoral Research Fellowship, Ms Orla McCourt, ICA-CDRF-2017-03-067). The views expressed in this publication are those of the author(s) and not necessarily those of the NHS, the National Institute for Health Research or the Department of Health and Social Care.

AUTHORS' CONTRIBUTIONS

OM drafted this manuscript, with input and final approval from KY, AF, GR, AR, JL, KR and NR. OM, KY, AF, GR designed and gained ethical approval for the original and amended study protocols. AR, JL, NR, KR provided intellectual input to the study protocols according to their area of expertise. All authors contributed to design of the study, conduct of the study, and are accountable for all aspects of the work.

ACKNOWLEDGMENTS

The authors wish to thank the people living with myeloma who provided input into the original and amended study designs.

COMPETING INTERESTS STATEMENT

None

1. Sathian, B., et al., *Impact of COVID-19 on clinical trials and clinical research: A systematic review.* Nepal J Epidemiol, 2020. **10**(3): p. 878-887.

For peer review only

2. Willan, J., et al., *Assessing the impact of lockdown: Fresh challenges for the care of haematology patients in the COVID-19 pandemic.* Br J Haematol, 2020. **189**(6): p. e224-e227.
3. Fersia, O., et al., *The impact of the COVID-19 pandemic on cardiology services.* Open Heart, 2020. **7**(2).
4. Meline, J., et al., *Adapting the design of a Web-based decision support clinical trial during the COVID-19 pandemic.* Trials, 2021. **22**(1): p. 734.
5. McCourt, O., et al., *PERCEPT myeloma: a protocol for a pilot randomised controlled trial of exercise prehabilitation before and during autologous stem cell transplantation in patients with multiple myeloma.* BMJ Open, 2020. **10**(1): p. e033176.
6. Bryant, M.S., S.E. Fedson, and A. Sharafkhaneh, *Using Telehealth Cardiopulmonary Rehabilitation during the COVID-19 Pandemic.* J Med Syst, 2020. **44**(7): p. 125.
7. HSE National Clinical Programme Respiratory, *Guidance for setting up Virtual Pulmonary Rehabilitation for Asthma and COPD during the Covid 19 pandemic*. 2020: Ireland.
8. Agency for Clinical Innovation, *Delivering pulmonary rehabilitation via telehealth during COVID-19*. 2020: Australia
9. Powell, L.E. and A.M. Myers, *The Activities-specific Balance Confidence (ABC) Scale.* J Gerontol A Biol Sci Med Sci, 1995. **50a**(1): p. M28-34.
10. Gephine, S., et al., *Cardiorespiratory Response during the 1-min Sit-to-Stand Test in Chronic Obstructive Pulmonary Disease.* Med Sci Sports Exerc, 2020. **52**(7): p. 1441-1448.

For peer review only

BMJ Open

Adaptation of the PERCEPT myeloma prehabilitation trial to virtual delivery: changes in response to the COVID-19 pandemic

Journal:	*BMJ Open*
Manuscript ID	bmjopen-2021-059516.R2
Article Type:	Communication
Date Submitted by the Author:	14-Mar-2022
Complete List of Authors:	McCourt, Orla; University College London Hospitals NHS Foundation Trust, ; University College London, Cancer Institute<br>Fisher, Abigail; University College London, Department of Epidemiology and Public Health<br>Ramdharry, Gita; National Hosital for Neurology and Neurosurger, Queen Square Centre for Neuromuscular Diseases; UCL, Deprtment of Neuromuscular Diseases<br>Roberts, Anna; University College London, Behavoural Science and Health<br>Land, Joanne; University College London, Behavoural Science and Health<br>Rabin, Neil; University College London Hospitals NHS Foundation Trust, Department of Haematology<br>Rowe, Katie; Univesrsity College London<br>Yong, Kwee; Univesrsity College London, Research Department of Haematology, Cancer Institute
**Primary Subject Heading**:	Haematology (incl blood transfusion)
Secondary Subject Heading:	Rehabilitation medicine
Keywords:	Myeloma < HAEMATOLOGY, Bone marrow transplantation < HAEMATOLOGY, COVID-19, Rehabilitation medicine < INTERNAL MEDICINE

SCHOLARONE™
Manuscripts

I, the Submitting Author has the right to grant and does grant on behalf of all authors of the Work (as defined in the below author licence), an exclusive licence and/or a non-exclusive licence for contributions from authors who are: i) UK Crown employees; ii) where BMJ has agreed a CC-BY licence shall apply, and/or iii) in accordance with the terms applicable for US Federal Government officers or employees acting as part of their official duties; on a worldwide, perpetual, irrevocable, royalty-free basis to BMJ Publishing Group Ltd ("BMJ") its licensees and where the relevant Journal is co-owned by BMJ to the co-owners of the Journal, to publish the Work in BMJ Open and any other BMJ products and to exploit all rights, as set out in our licence.

The Submitting Author accepts and understands that any supply made under these terms is made by BMJ to the Submitting Author unless you are acting as an employee on behalf of your employer or a postgraduate student of an affiliated institution which is paying any applicable article publishing charge ("APC") for Open Access articles. Where the Submitting Author wishes to make the Work available on an Open Access basis (and intends to pay the relevant APC), the terms of reuse of such Open Access shall be governed by a Creative Commons licence – details of these licences and which Creative Commons licence will apply to this Work are set out in our licence referred to above.

Other than as permitted in any relevant BMJ Author's Self Archiving Policies, I confirm this Work has not been accepted for publication elsewhere, is not being considered for publication elsewhere and does not duplicate material already published. I confirm all authors consent to publication of this Work and authorise the granting of this licence.

For peer review only

Adaptation of the PERCEPT myeloma prehabilitation trial to virtual delivery: changes in response to the COVID-19 pandemic

Corresponding Author:

Orla McCourt

Department of Haematology, UCL Cancer Institute, University College London, UK & University College London Hospitals NHS Foundation Trust, London, UK

Postal Address: Therapy Office, Lower Ground Floor (T-1), University College Hospital, 235 Euston Road, London NW1 2BU, UK

Email: o.mccourt@nhs.net

Telephone: 020 7679 1736

Authors:

Orla McCourt – Therapies & Rehabilitation, University College London Hospitals NHS Foundation Trust, London, UK; Research Department of Haematology, Cancer Institute, University College London, London, UK

Abigail Fisher - Research Department of Behavioural Science and Health, University College London, London, UK

Gita Ramdharry - Queen Square MRC Centre for Neuromuscular Diseases, Institute of Neurology, University College London, London, UK

Anna Roberts - Research Department of Behavioural Science and Health, University College London, London, UK

Joanne Land - Research Department of Haematology, Cancer Institute, University College London, London, UK

Neil Rabin – Department of Haematology, University College London Hospitals NHS Foundation Trust, London, UK

Katie Rowe - Research Department of Haematology, Cancer Institute, University College London, London, UK

Kwee Yong - Research Department of Haematology, Cancer Institute, University College London, London, UK

Word Count: 2179

For peer review only

ABSTRACT

Introduction and objective Research activity was impacted by the novel coronavirus disease (COVID-19) pandemic, the PERCEPT myeloma trial was no exception. This pilot randomised trial delivered a face-to-face exercise intervention prior to and during autologous stem cell transplantation (ASCT) in myeloma patients, as a consequence of COVID-19 it required significant adaptions to continue. This brief communication describes how the previously published study protocol was adapted for virtual delivery. In addition, we highlight the challenge of continuing the study which was embedded within a clinical pathway also impacted by the pandemic.

Summary The original trial protocol was amended and continued to recruit and deliver an exercise prehabilitation intervention virtually. Continued delivery of the intervention was deemed important to participants already enrolled within the trial and the adapted virtual version of the trial was acceptable to the research ethics committee as well as participants. Development of effective, remotely delivered rehabilitation and physical activity programmes are likely to benefit people living with myeloma. The COVID-19 pandemic provided an opportunity to explore the feasibility of a virtual programme for ASCT recipients, however continued changes to the clinical pathway within which the study was embedded posed the greatest challenge and ultimately led to early termination of recruitment.

For peer review only

INTRODUCTION

The threat of the novel coronavirus disease (COVID-19) and declaration of a pandemic greatly impacted research activity around the world with many studies and trials paused and recruitment ceased unless COVID-19 related or providing potential life preserving interventions[1]. Pandemic responses led to changes in how clinical services were delivered across health systems, with many usual face to face outpatient activities changing quickly to be remotely delivered via telephone or video[2].

Reports have detailed how clinical services and clinical trials adapted in response to the need to lower the threat posed by COVID-19[3,4], initially through reduced face to face attendance at healthcare institutions and protecting the most clinically vulnerable by promoting minimal activity outside of the home, practising social distancing and discouraging use of public transport[5]. In the United Kingdom, people considered to be clinically extremely vulnerable, such as those with haematological cancers, were advised to shield to protect themselves from exposure to infection. As a result, clinical pathways and research protocols required significant adaptations to continue[6-8]. We report the challenges of continuing and adapting a prehabilitation and rehabilitation exercise intervention trial in response to the COVID-19 outbreak and subsequent changes to the clinical pathway into which the trial was integrated. This communication seeks to document changes to our previously published protocol, as well as facilitate study design in the immediate post pandemic era where future interventional trials need to be adaptable in case of unexpected future pandemics or other health emergencies.

BACKGROUND TO TRIAL

The PERCEPT myeloma prehabilitation study was a pilot, single-centre, randomised controlled trial (RCT) that aimed to investigate the feasibility of a physiotherapist-led exercise intervention, designed to be embedded as an integral part of an established clinical pathway for patients with multiple myeloma undergoing autologous stem cell transplantation (ASCT). Recruitment to the trial commenced at University College Hospital London (UCLH) in June 2019. In summary, all patients referred to the centre for consideration of ASCT for management of myeloma, who were willing to and clinically able to undertake an exercise training programme and had good command of written and spoken English were eligible and approached. The trial assessments included a range of objective measures of functional capacity (six minute walk test, 30 second timed sit to stand test, hand grip strength test, levels of physical activity) as well as patient reported outcome measures assessing quality of life (QOL) (Functional Assessment of Cancer Therapy (FACT) bone marrow transplant and European Organisation for Research and Treatment of Cancer QLQ-C30), fatigue (FACT fatigue subscale) and physical activity behaviour (International Physical Activity Questionnaire and exercise

For peer review only

self-efficacy scale). Assessments were initially designed to be conducted face-to-face at established touchpoints within the clinical pathway that usually coincided with patient attendance at transplant clinic appointments. The intervention involved an exercise intervention with incorporated behaviour change techniques, delivered in part through weekly face-to-face, group-based exercise sessions at a UCLH gym supervised by a physiotherapist and through unsupervised home exercise sessions with telephone support in the post-ASCT rehabilitation phase. Participants were asked to exercise three times per week, at moderate to high intensity, for 30 minutes of aerobic exercise and complete a programme of resistance exercises. Exercise was individually tailored and progressed by the study physiotherapist. In the prehabilitation phase before ASCT participants were expected to attend weekly supervised gym sessions and exercise independently twice per week. During hospital admission and following discharge from hospital participants were supported to continue three unsupervised exercise sessions per week with weekly telephone guidance from a physiotherapist. The original trial was registered (ISRCTN15875290) and the protocol is described in detail elsewhere[9].

TRIAL RECRUITMENT PRIOR TO THE PANDEMIC

Trial recruitment was progressing as planned and between June 2019 and end of February 2020 90 potential participants had been identified and screened for eligibility. Of those, n=36 (40%) consented to take part and were randomised, n=8 (9%) were ineligible and n=46 (51%) declined. The main reasons for declining to take part were travel or distance to take part in the intervention, poor mobility/inability to use public transport and already having too many hospital appointments. Many of those who declined had a favourable opinion of the trial and would have taken part if it was closer to their home. It had been anticipated that target recruitment (n=60-75) would be reached by December 2020.

Face to face recruitment was paused in March 2020, due to the COVID-19 pandemic, when all but essential NHS research activity was instructed to cease and research physiotherapists were redeployed to clinical services. In addition, the myeloma ASCT service was also paused due to resource limitations, lack of critical care provision and increased risk of COVID-19 infection in transplant recipients.

RESUMING RECRUITMENT AND ADAPTATION TO VIRTUAL INTERVENTION DELIVERY

Between March and June 2020, all participants already enrolled within the trial expressed their wish to continue in the trial whilst awaiting their delayed ASCT. All participants were placed on holding chemotherapy by their clinical teams. A minor ethics amendment was granted to allow participants to remain in the trial longer than in the original protocol, to continue delivery of the home-based

For peer review only

component of the exercise intervention via telephone support and to collect follow up assessment data via postal delivery of trial questionnaires.

Following resumption of the myeloma ASCT clinical service in June 2020, elements of the clinical pathway on which the trial procedures were designed had changed significantly in response to the ongoing threat of the pandemic. Namely, patients were no longer attending UCLH clinics in person in the lead up to their ASCT and were instead receiving telephone contact for clinic appointments. It was therefore necessary to adapt the trial to accommodate these changes to the clinical pathway.

Patient and public involvement

Participants who had remained in the study during the first national lock down were consulted on how best to proceed with the trial. These participants were asked to consider their acceptability of receiving study assessments/questionnaires via post, and importantly, how they would feel about only having access to the physiotherapist and exercise intervention via video platform. Overwhelmingly, they supported adapting the trial to virtual delivery and reported that having regular contact with the study physiotherapist was supportive, especially as they were no longer attending their clinical services in person. Some reported that the exercise intervention was something positive to focus on while feeling apprehensive about having their ASCT delayed and remaining limited in their social activities due to shielding. These participants were consulted on the proposed substantial amendment and gave their approval to the changes described in this manuscript.

Ethical approval

Following review of emerging literature for adapting pulmonary rehabilitation services to virtual delivery[10-12] and liaising with other cancer rehabilitation services delivering exercise virtually, the trial design was substantially amended. A substantial amendment was approved by the ethics committee (London - Camden & Kings Cross reference 19/LO/0204) to continue the trial with virtual enrolment and consent process, baseline assessment and delivery of a group-based intervention via the online platform Zoom, described below.

Virtual recruitment, consenting and baseline assessments

- Identifying potential participants from a list of patients awaiting consideration for ASCT, rather than from weekly clinic lists of expected clinic attendance.
- An additional inclusion criterion was added to require patients to have access to and the ability to use internet-based video conferencing platform, or a family member to facilitate access.

For peer review only

- Participants were sent trial consent forms, self-complete questionnaire measures and a pre-programmed ActivPAL accelerometer by post and a Zoom video call appointment was arranged for approximately 3 days later. Completion of consent forms, baseline questionnaires and fitting of accelerometer discussed and conducted virtually over Zoom appointment.
- Addition of the Activities-Specific Balance Confidence Scale[13] to assess risk of falling, used to screen those randomised to the intervention for any necessary actions required to reduce risk of exercising at home as no longer objectively assessed by the physiotherapist face to face. If any participant scored <65% on the scale they would be deemed high risk of falling[14] and their assessment and participation in the group sessions would be adapted to minimise risk.
- Objective measure of functional capacity changed to a remotely delivered one minute timed sit to stand test, which has been demonstrated to induce comparable cardiovascular stress and lower limb muscle fatigue as the six minute walk test[15].

Virtual delivery of the partly supervised exercise intervention

- The intervention delivery was changed to be completely online but following the principles of the original protocol in terms of frequency, intensity, time and type of exercise. Each new participant had an initial one to one session to introduce the programme and tailor to their individual abilities, including any symptoms of bone disease or balance deficits. Each participant was then asked to attend a weekly virtual group-based exercise class via Zoom until they were admitted for their ASCT.

RECRUITMENT TO VIRTUAL TRIAL TO DATE

Recruitment restarted after the first wave of the pandemic in August 2020. Up to October 2020 a further 33 potential participants were identified and screened for eligibility. Of those, 14 (43%) agreed and consented to take part, 6 (18%) were ineligible and 13 (39%) declined. This indicates that uptake to the virtual part of the trial was similar to the original face to face design. Despite fewer people declining the virtual trial, more people were ineligible. The main reason for increased proportion of ineligibility was related to some potential participants already having a date to be admitted for ASCT within the succeeding 2-4 weeks or not having access to the internet.

With the resurgence of COVID-19 in the Autumn of 2020 and as clinical services for ASCT were disrupted again, it was decided to cease recruitment to the trial. The predominant challenge to the adapted trial was variable intervals between timepoints, which differed in length from those detailed in the original study schedule. Further, a small number of participants were withdrawn from the trial

For peer review only

as their planned ASCT was deferred or delayed due to clinical risk of proceeding with the ongoing threat of the COVID-19 virus and prioritisation of ASCT admissions based on clinical need in this context. The main aim of the study was to investigate the feasibility of delivering an intervention embedded within an existing clinical pathway. As the pathway upon which the trial was designed was significantly affected by the Covid-19 pandemic, which would likely continue to affect the pathway for some time, it was agreed by the study team that little more would be gained by recruiting further. The trial continued in follow up until all those recruited either reached the final timepoint following ASCT or were withdrawn. The challenges to timely recruitment and delivery of the trial timepoints as based on the clinical ASCT pathway have implications on study design and will be addressed with the full reporting of the trial which is expected in 2022.

CONCLUSION

The emergence of the COVID-19 pandemic required significant adaptation for the continuation of this pilot RCT. Our sample population being clinically extremely vulnerable were advised to shield, as well as the requirement to reduce all but clinically necessary face to face contact altered how potential ASCT recipients accessed the clinical setting and consequently the planned trial delivery. We have briefly described how the trial protocol was amended and continued to recruit to and deliver a prehabilitation intervention virtually. Adaptation to virtual delivery was deemed important to participants already enrolled within the trial and appears to have been acceptable for the adapted virtual version of the trial while this was possible.

The original trial design relied heavily on participant attendance at face to face clinical contacts as part of the routine ASCT pathway as it existed pre-pandemic. This was a strength in terms of increasing likely participation in follow up study assessments and ensuring completeness of data capture as participants would be present in the centre at the planned follow up timepoints. However, the assumption that all assessments would be conducted face to face resulted in a reliance on paper-based questionnaires and because study resources did not allow for transformation to electronic data capture, postal follow up assessment was required. On reflection, the original study would have been somewhat future proofed had electronic questionnaires and intervention materials been incorporated at design and should certainly be a requirement of future work.

As ASCT recipients with myeloma are generally older, with high incidence of disease-related bone disease, deconditioned by induction chemotherapy with possible impairments to mobility and function, adapting the trial to explore feasibility of a virtually delivered physiotherapy intervention was an important opportunity arising from the threat posed by the pandemic. Considering the possible increased risk from not assessing participants face to face, required implementation of a remotely

For peer review only

delivered balance assessment tool, an alternate objective measure of functional capacity suitable for remote assessment, and on-line adaptation of intervention delivery to include a virtual group-based exercise class were all important steps. Development of effective, remotely delivered rehabilitation and physical activity programmes are likely to be increasingly valuable to people living with myeloma for whom shielding during clinically vulnerable periods of treatment and recovery, and travel to their specialist treatment centres will be continuing challenges in the post pandemic era.

FUNDING STATEMENT

This communication relates to independent research supported by the National Institute for Health Research (HEE/ NIHR ICA Programme Clinical Doctoral Research Fellowship, Ms Orla McCourt, ICA-CDRF-2017-03-067). The views expressed in this publication are those of the author(s) and not necessarily those of the NHS, the National Institute for Health Research or the Department of Health and Social Care.

AUTHORS' CONTRIBUTIONS

OM drafted this manuscript, with input and final approval from KY, AF, GR, AR, JL, KR and NR. OM, KY, AF, GR designed and gained ethical approval for the original and amended study protocols. AR, JL, NR, KR provided intellectual input to the study protocols according to their area of expertise. All authors contributed to design of the study, conduct of the study, and are accountable for all aspects of the work.

ACKNOWLEDGMENTS

The authors wish to thank the people living with myeloma who provided input into the original and amended study designs.

COMPETING INTERESTS STATEMENT

None

For peer review only

References

1. Sathian, B., et al., *Impact of COVID-19 on clinical trials and clinical research: A systematic review.* Nepal J Epidemiol, 2020. **10**(3): p. 878-887.
2. Gilbert, A.W., et al., *Rapid implementation of virtual clinics due to COVID-19: report and early evaluation of a quality improvement initiative.* BMJ Open Qual, 2020. **9**(2).
3. Mitchell, E.J., et al., *It is unprecedented: trial management during the COVID-19 pandemic and beyond.* Trials, 2020. **21**(1): p. 784.
4. NHS Providers, *Confronting coronavirus in the NHS: the story so far*. 2020: Online.
5. Dunn, P., et al., *COVID-19 policy tracker*. 2020: Online.
6. Willan, J., et al., *Assessing the impact of lockdown: Fresh challenges for the care of haematology patients in the COVID-19 pandemic.* Br J Haematol, 2020. **189**(6): p. e224-e227.
7. Fersia, O., et al., *The impact of the COVID-19 pandemic on cardiology services.* Open Heart, 2020. **7**(2).
8. Meline, J., et al., *Adapting the design of a Web-based decision support clinical trial during the COVID-19 pandemic.* Trials, 2021. **22**(1): p. 734.
9. McCourt, O., et al., *PERCEPT myeloma: a protocol for a pilot randomised controlled trial of exercise prehabilitation before and during autologous stem cell transplantation in patients with multiple myeloma.* BMJ Open, 2020. **10**(1): p. e033176.
10. Bryant, M.S., S.E. Fedson, and A. Sharafkhaneh, *Using Telehealth Cardiopulmonary Rehabilitation during the COVID-19 Pandemic.* J Med Syst, 2020. **44**(7): p. 125.
11. HSE National Clinical Programme Respiratory, *Guidance for setting up Virtual Pulmonary Rehabilitation for Asthma and COPD during the Covid 19 pandemic*. 2020: Ireland.
12. Agency for Clinical Innovation, *Delivering pulmonary rehabilitation via telehealth during COVID-19*. 2020: Australia
13. Powell, L.E. and A.M. Myers, *The Activities-specific Balance Confidence (ABC) Scale.* J Gerontol A Biol Sci Med Sci, 1995. **50a**(1): p. M28-34.
14. Lajoie, Y. and S.P. Gallagher, *Predicting falls within the elderly community: comparison of postural sway, reaction time, the Berg balance scale and the Activities-specific Balance Confidence (ABC) scale for comparing fallers and non-fallers.* Arch Gerontol Geriatr, 2004. **38**(1): p. 11-26.
15. Gephine, S., et al., *Cardiorespiratory Response during the 1-min Sit-to-Stand Test in Chronic Obstructive Pulmonary Disease.* Med Sci Sports Exerc, 2020. **52**(7): p. 1441-1448.

For peer review only
